# Supplementary material for: The hEag1 K+ Channel Inhibitor Astemizole Stimulates Ca2+ Deposition in SaOS-2 and MG-63 Osteosarcoma Cultures
Source: Int J Mol Sci. 2022 Sep 11;23(18):10533. doi: 10.3390/ijms231810533 (PMC9504018; doi:10.3390/ijms231810533)
Supplement: Supplementary file 1 [file ijms-23-10533-s001.zip › ijms-1873107-supplementary.pdf]

The hEag1 K^+ channel inhibitor astemizole stimulates Ca^{2+} deposition in SaOS-2 and MG-63 osteosarcoma cultures

Beáta Mészáros[1,2], Agota Csoti[1], Tibor G. Szanto[1], Andrea Telek[1,3], Katalin Kovács[4], Agnes Toth[1,5], Julianna Volkó[1] and Gyorgy Panyi[1,*]

Supplemental material

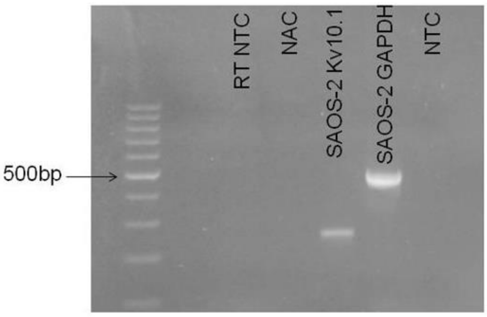

RT NTC: no template control in the reverse transcription
NAC: no amplification control
NTC: no template control in PCR
Kv10.1 primers:
F1: 5' TCT GTC TAC ATC TCC TCG TT 3'
R1: 5' CCA TTA CTC GCT CAC TCA 3'

Supplementary Figure S1. hEag1(Kv10.1) is detected at mRNA level in SaOS-2 cells. Identification of Kv10.1 mRNA was carried out by means of RT-PCR, using intron spanning primers. Kv10.1 PCR products are 271 bp (shown as SAOS-2 Kv10.1 on the gel electrophoresis photo). Reverse transcription was validated using RT NTC (non-template control in reverse transcription) and NAC (no revers transcription control). Genomic Kv10.1 DNA was not amplified in the Kv10.1 positive samples, as shown by the NAC (no revers transcription control) sample. PCR was validated using GAPDH positive (PCR product is 496 bp, shown as SAOS-2 GAPDH in the figure) and NTC (non-template control) control. The experiment was repeated n=3 times.

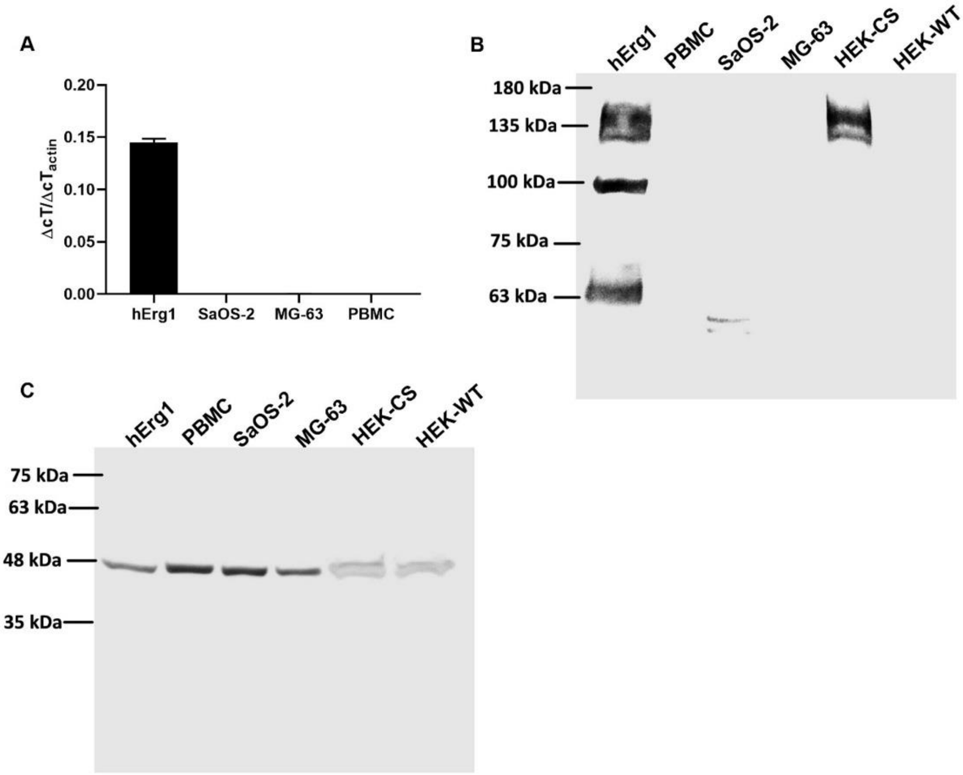

Supplementary Figure S2. Analysis of hErg1 channel expression using QPCR and Western blotting in SaOS-2 and MG-63 cells prior to differentiation induction. (**A**) QPCR data in the first column show the expression level of the hErg1 mRNA in HEK 293 cells stably expressing the hErg1 gene (as a positive control), the second and third columns show it in SaOS-2 and MG-63 cells, and the last column presents the quantification of hErg1 transcripts in Peripheral Blood Mononuclear Cells (PBMC, negative control). GAPDH was used as the internal standard. **(B)** Immunoreactivity of the anti-hErg1 antibody. hErg1: proteins were extracted from HEK 293 cells stably expressing hErg1 gene (as a positive control); PBMC: proteins were extracted from PBMC (as negative control); SaOS-2: proteins were extracted from SaOS-2 cells; MG-63: proteins were extracted from MG-63 cells; HEK-CS: lysates from HEK 293 cells stably expressing hErg1 gene (as a positive control, from A. Arcangeli); HEK-WT: lysates from HEK 293 cells (as a negative control, from A. Arcangeli). The anti-hErg1 antibodies reveal specific bands only in the two positive controls, with a predicted molecular mass of 130-150 kDa (hErg1 isoform). **(C)** Immunoreactivity of the anti-actin antibody. The scheme is the same as previously. The anti-actin antibodies reveal specific bands in all samples with a predicted molecular mass of 42 kDa.

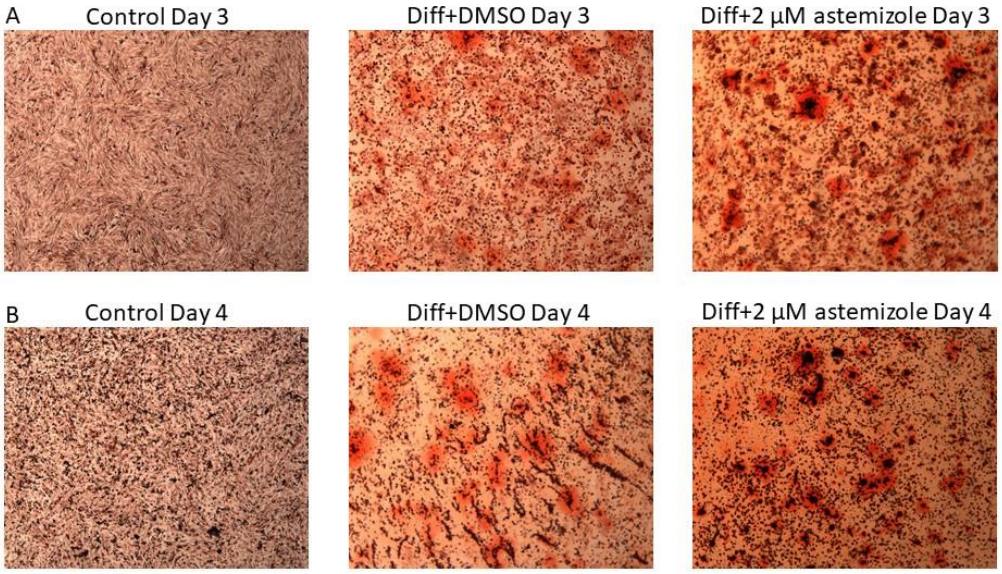

Supplementary Figure S3. Pathological pathway (Pi)-induced Ca^{2+} deposition in SaOS-2 cultures in the presence of astemizole. For the evaluation of matrix mineralization cells were stained with Alizarin Red S (stains calcium). Alizarin Red S-stained cultures were photographed with a microscope equipped with a digital camera. The hydroxyapatite containing bone nodules are recognizable with red color. The photos were taken on Day 3 **(A)** and Day 4 **(B)** as indicated. Labels indicate SaOS-2 cell cultures treated with Control: no differentiation cocktail; Diff+DMSO: Pathological pathway (Pi)-induced mineral matrix production of SaOS-2 cells using DMSO control. Diff+2 μM astemizole: Pathological pathway (Pi)-induced mineral matrix production of SaOS-2 cells in the presence of 2 μM astemizole.

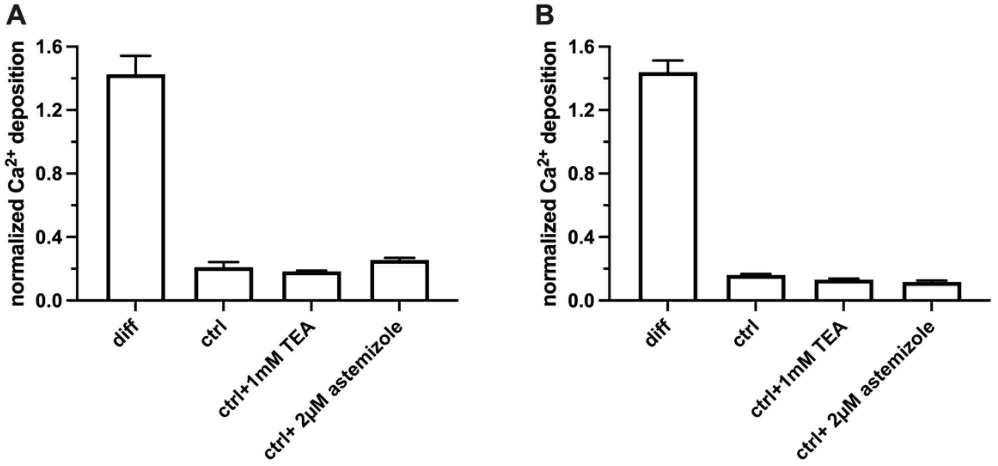

Supplementary Figure S4. Ca^{2+} deposition in SaOS-2 cultures in the presence of hEag1 inhibitors but in the absence of differentiation inducing cocktail. The inhibitors were added to the cell cultures at the beginning of osteogenic induction. The Ca^{2+} deposits were detected using Alizarin Red staining and quantification was achieved by dissolving Alizarin Red –calcium complexes in CPC and measuring absorbance (see Methods). Labels indicate SaOS-2 cell cultures treated with ctrl: no differentiation cocktail; diff: mineralization induced by differentiation cocktail; ctrl+1 mM TEA: no differentiation cocktail but treatment with 1 mM TEA; ctrl+2 μM astemizole: no differentiation cocktail but treatment with 2 μM astemizole. Staining was done on Day 4 **(A)** and Day 7 **(B)**. Graphs shows only a representative experiment (4-4 samples/column).

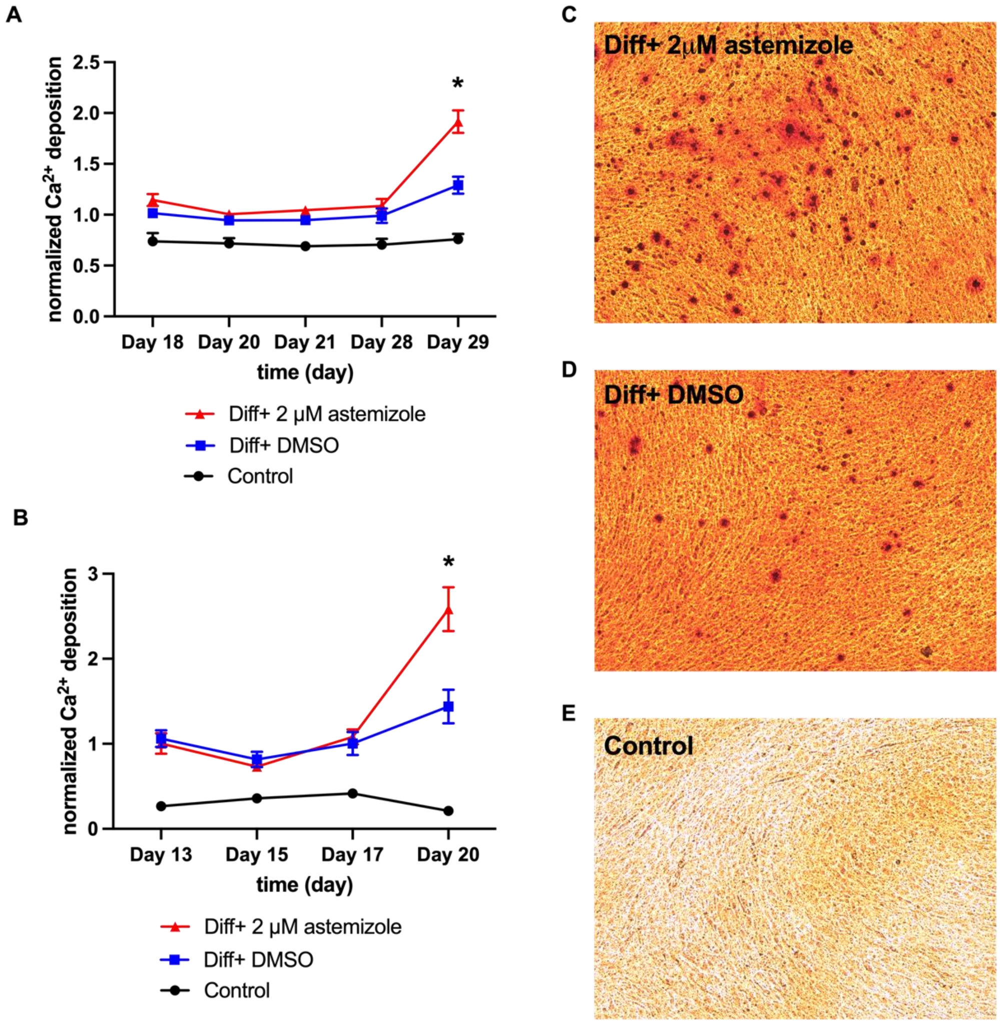

Supplementary Figure S5. Ca^{2+} deposition in MG-63 cultures in the presence of astemizole. (A) Classical pathway-induced Ca^{2+} depositions in MG-63 cells in the presence and absence of astemizole. Astemizole (2 μM) was added to the cell cultures at the beginning of osteogenic induction. M The Ca2+ deposits were detected using Alizarin Red staining and quantification was achieved by dissolving Alizarin Red –calcium complexes in CPC and measuring ab-sorbance (see Methods). Normalized Ca2+ deposition was calculated as A/A_{PBS} where A is the absorbance of a given sample and APBS is the average absorbance of differentiation-induced cells in the presence of PBS (vehicle control; data not shown on the graph), respectively. Labels indicate MG-63 cultures treated with Control: no differentiation cocktail; diff+DMSO: differentiation induced by the classical pathway and added DMSO as vehicle control for astemizole; diff+2 μM astemizole: differentiation induced by classical pathway of osteogenesis and treated with 2 μM astemizole. Data were obtained on Day 18, Day 20, Day 21, Day 28, Day 29. Data are presented as mean SEM (n > 4) and analyzed using One Way RM ANOVA statistical test, * $p<0.05$. (B) Pathological pathway (Pi)-induced Ca^{2+} depositions in MG-63 cultures in the presence and absence of astemizole. Mineralization of MG-63 cells was induced via the pathological pathway using inorganic phosphate (Pi). For all other details see the legend to panel A. Labels indicate MG-63 cell cultures treated with Control: no differentiation induction by Pi; diff+DMSO: differentiation induced by Pi and added DMSO as vehicle control for astemizole; diff+2 μM astemizole: differentiation induced by Pi and treated with 2 μM astemizole. Data are presented as mean±SEM (n > 4) and analyzed using One Way RM ANOVA statistical test, * $p<0.05$. (C) Pathological pathway (Pi)-induced Ca^{2+} depositions in MG-63 cultures in the presence of DMSO (diff+DMSO). For the evaluation of matrix mineralization cells were stained with Alizarin Red S (stains calcium). Alizarin Red S-stained cultures were photographed with a microscope equipped with a digital camera. The photos were taken on Day 20. (D) Pathological pathway (Pi)-induced Ca^{2+} depositions in MG-63 cultures in the presence of 2 μM astemizole (diff+2 μM astemizole). Alizarin Red S-stained cultures were photographed on Day20. The hydroxyapatite containing bone nodules are recognizable with red color. (E) MG-63 cells without differentiation induction (Control). Alizarin Red S-stained cultures were photographed as the above-mentioned manner on Day 20.

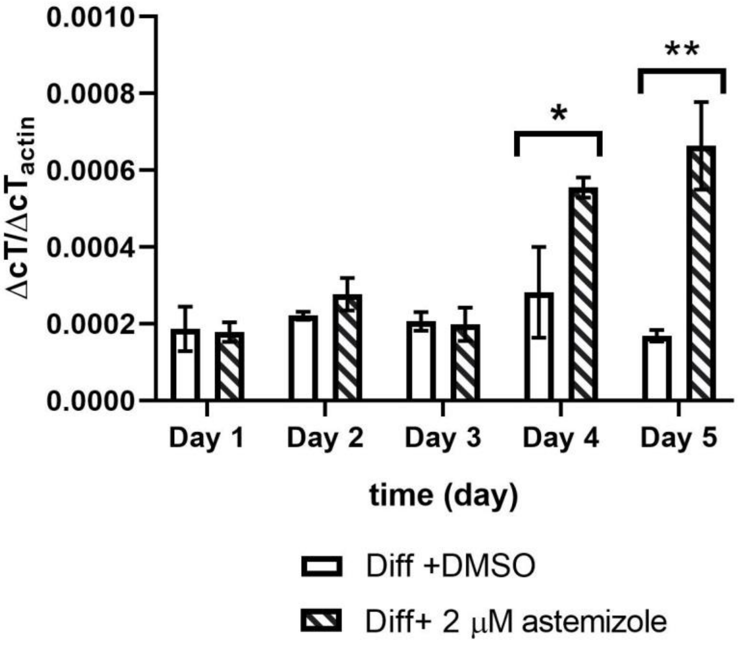

Supplementary Figure S6. Quantification of hEag1 mRNA expression during mineralization in SaOS-2 cells using QPCR. We followed up the expression hEag1 mRNA in the astemizole-treated samples (2 μM, hatched columns) and the DMSO-treated samples (empty columns) on five different days (on Day1, Day 2, Day3, Day 4 and Day 5, as indicated). Data are presented as mean±SD (n=3) and analyzed t-test using test, * $p<0.05$; ** $p<0.001$
